# *Heterodera avenae* GLAND5 Effector Interacts With Pyruvate Dehydrogenase Subunit of Plant to Promote Nematode Parasitism

**DOI:** 10.3389/fmicb.2019.01241

**Published:** 2019-06-04

**Authors:** Shanshan Yang, Lingling Pan, Yongpan Chen, Dan Yang, Qian Liu, Heng Jian

**Affiliations:** Department of Plant Pathology and MOA Key Laboratory of Pest Monitoring and Green Management, China Agricultural University, Beijing, China

**Keywords:** *Heterodera avenae*, effector, HaGland5, pyruvate dehydrogenase subunit, defense signaling pathways

## Abstract

*Heterodera avenae* mainly infects cereal crops and causes severe economic losses. Many studies have shown that parasitic nematodes can secrete effector proteins to suppress plant immune responses and then promote parasitism. In this study, we showed that HaGland5, a novel effector of *H. avenae*, was exclusively expressed in dorsal esophageal gland cell of nematode, and up-regulated in the early parasitic stage. Transgenic *Arabidopsis thaliana* lines expressing *HaGland5* were significantly more susceptible to *H. schachtii* than wild-type control plants. Conversely, silencing of *HaGland5* through barley stripe mosaic virus-medicated host-induced gene silencing technique substantially reduced the infection of *H. avenae* in wheat. Moreover, HaGland5 could suppress the plant defense responses, including the repression of plant defense-related genes, reducing deposition of cell wall callose and the burst of reactive oxygen species. Mass spectrometry, co-immunoprecipitation, and firefly luciferase complementation imaging assays confirmed that HaGland5 interacted specifically with *Arabidopsis* pyruvate dehydrogenase subunit (AtEMB3003).

## Introduction

The cereal cyst nematode, *Heterodera avenae*, can infect wheat, barley, and oat crops in most cereal-growing regions of the world, it is an economically important nematode (Bonfil et al., 2004). Hatched second stage juveniles (J2s) penetrate their host plant roots and then migrate intracellularly toward the vascular cylinder, where they chose a special cell that is suitable to establish the initial syncytial cell (Wyss and Zunke, 1986; Wyss and Grundler, 1992). From this, a multinucleate syncytium develops through the fusion of neighboring cells (Grundler et al., 1998). Cyst nematodes are obligate sedentary endoparasites, and the syncytium is the only source of nutrients for the nematode development; therefore, they interact with their host plants closely until their reproduction is complete. To parasitize a host plant successfully, nematodes secrete effector proteins into plant cells via a hollow stylet, which suppress plant defenses and alter developmental and physiological processes (Gheysen and Mitchum, 2011).

Plants have developed a two-layer immune system to protect them from pathogen attacks (Jones and Dangl, 2006), including pathogen-associated molecular pattern triggered immunity (PTI) and effector triggered immunity (ETI). Plant pattern recognition receptors can recognize conserved pathogen molecules, triggering callose deposition, the burst of reactive oxygen species, and expression of defense-related genes, which is PTI (Luna et al., 2011; Mendoza, 2011); ETI is that plant R proteins recognize effector proteins of pathogens, causing a hypersensitive cell death response localized in the infection site to restrict the spread of the pathogen (Heath, 2000). In recent years, it has been found that many effectors secreted by phytopathogens, such as bacteria, fungi, and oomycetes, could suppress immune signaling and promote parasitism (Dou and Zhou, 2012). There is emerging evidence that effectors secreted by cyst nematodes also play an important role in modulating plant immune responses, such as GrSPRYSEC19 (Postma et al., 2012), GrCEP12 (Chronis et al., 2013), GrVAP1 (Lozano-Torres et al., 2014), Ha-ANNEXIN (Chen et al., 2015), and HgGLAND18 (Noon et al., 2016).

*Arabidopsis thaliana* is a model plant in the mustard family Brassicaceae (Meinke et al., 1998), and it has been used to explore the interaction between nematodes and plants, such as in root-knot nematodes (Schechter et al., 2006; Canonne et al., 2011; Gheysen and Fenoll, 2011; Lin et al., 2016; Niu et al., 2016; Germain et al., 2018). However, for cyst nematodes, only *H. schachtii* can parasitize and reproduce on *A. thaliana* (Sijmons et al., 1991; Fmw et al., 1994). In the last few years, using the *H. schachtii–A. thaliana* pathosystem, some effectors of cyst nematodes have been functionally analyzed (Hewezi et al., 2008, 2010; Pogorelko et al., 2016; Barnes et al., 2018). For example, to aid functional characterization of Hg25A01, an esophageal gland cell effector from *H. glycines*, *Hs25A01* from the closely related *H. schachtii* was cloned. Constitutive expression of *Hs25A01* led to increased susceptibility to *H. schachtii* in *A. thaliana*, indicating that Hg25A01 also promoted nematode parasitism (Pogorelko et al., 2016). This supports the feasibility of using the *H. schachtii*–*A. thaliana* pathosystem to explore the effector function of other cyst nematodes. The genome of wheat is complex, which reduces the efficacy of transgenic studies; therefore, the *H. schachtii*–*A. thaliana* pathosystem may be a useful tool to explore the function of *H. avenae* effectors.

In plants, fatty acids are important in a diverse range of biological processes (Shanklin and Cahoon, 1998; Maldonado et al., 2002; Sumin et al., 2010), such as the regulation of various plant defense signaling pathways (Kachroo et al., 2003). Plastidial fatty acids, unlike mitochondrial counterparts, can modulate defense signaling pathway mediated by salicylic acid and jasmonic acid (Kachroo et al., 2003; Chandra-Shekara et al., 2007). For the biosynthesis of plastidial fatty acids, plastid pyruvate dehydrogenase complex (PDC) provides the fatty acid precursor acetyl-CoA (Johnston et al., 1997), which is involved in many metabolic pathways, such as glycolysis and tricarboxylic acid cycles.

The first comprehensive parasitome profile of *H. glycines* was obtained by analyzing the cDNA library of gland cells. Among them, G16B09 and 4D06 effectors and related proteins (herein referred to as the “G16B09 family”) were initially identified in *H. glycines* (Gao et al., 2003). Up to date, 11 numbers from *H. glycines* had been identified (Gao et al., 2003; Noon et al., 2015). The G16B09 family was not only found in *H. glycines*, it was also considered to be one of the largest families in *G. pallida* with 39 members identified (Thorpe et al., 2014). All the mRNAs of G16B09 family effectors are expressed in the dorsal esophageal gland cell, indicating that this family may contribute to the induction of syncytium. Moreover, they are novel transcripts without any homolog in public databases (Yang et al., 2019). Exploring the function of G16B09 family effector may provide evidences for better understanding nematode-plant interactions. In this study, we identified a new effector HaGland5 belonging to G16B09 family from *H. avenae*, which owned all the characteristic of this family. Furthermore, HaGland5 could suppress plant defense and promotes parasitism by modulating defense signaling pathways in plants.

## Materials and Methods

### Nematode and Plant Materials

The cysts (*H. avenae*) were collected from Qingdao, China, stored at least 4 weeks at 4°C before hatching. The pre-parasitic second stage juveniles (pre-J2s) were obtained by hatching the cysts at 15°C. To obtain nematodes in different stages, the whole infected wheat roots were collected at 5, 20, and 30 days post inoculation (dpi), cut into sections, and then digested in a 6% cellulose solution overnight by shaker at 160 rpm/min and 28°C. *H. schachtii* were propagated on beets (*Beta vulgaris* L.), and pre-J2s were collected by hatching the cysts at 25°C.

Wheat seedlings (*Triticum. aestivum* cv. Aikang 58) or *Nicotiana benthamiana* seedlings were grown in a growth chamber at 22°C with a 16 h light/8 h dark cycle or at 25°C with a 14 h light/10 h dark cycle, respectively. Surface-sterilized *Arabidopsis thaliana* was sown on Murashige and Skoog solidified (MS) medium under sterile condition, then the seedlings were transplanted into potting soil in a growth chamber with 16 h light/8 h dark cycle at 23°C.

### Sequence Analysis

To obtain the homologous genes of *HaGland5* from *H. glycines*, *G. pallida*, and *G. rostochiensis*, a BLAST search against the public genome database was performed (Cotton et al., 2014; Eves-van den Akker et al., 2016). The homologous sequence of *H. schachtii* was obtained by amplification using the primers *HgGland5*-F/*HgGland5*-R (Supplementary Table S1). The sequence homology of these proteins, the conserved domains, the signal peptide of effectors, putative transmembrane domains and the subcellular localization *in planta* was analyzed by DNAMAN, NCBI CD-Search^1^, SignalP 4.1^2^, TMHMM^3^, and PSORT^4^, respectively. And MEGA6.0 was used to build phylogenetic trees using the Neighbor-Joining method.

### Expression Analysis

For *in situ* hybridization, pre-parasitic *H. avenae* nematodes hatched in leachates of wheat root were used, because preliminary tests showed that no signals were detected in pre-J2s hatched in water. The DIG-labeled sense and antisense cDNA probes (Roche, United States) were synthesized by an asymmetric PCR, using primers *in-situ-HaGland5*-F/*in-situ-HaGland5*-R (Supplementary Table S1). Hybridization experiment was performed as described previously (Smant, 1998), and examined under a BX51 microscope (Olympus, Japan). For observing the position of one dorsal esophageal gland cell and two subventral esophageal gland cells of *H. avenae*, we collected some J2s and killed them by heating 65°C 2 min, and then the photos were taken using same microscope.

For developmental expression level assay, total RNA of *H. avenae* in different life stages (including egg, pre-J2s, par-J2s, J3s, J4s, and females) was extracted using RNeasy Plus Micro Kit (Qiagen, Germany). M-MLV (Takara, Tokyo, Japan) was used to obtain gDNA free’s nematode cDNA according to the manufacturer’s instructions. SYBR Premix Ex Taq II (Tli RNaseH Plus; Takara, Tokyo, Japan), the primers *HaGland5-*qRT-F/*HaGland5*-qRT-R and *GAPDH-1*-qRT-F/*GAPDH-1*-qRT-R, which were derived from the *HaGland5* gene and the reference gene *GAPDH-1* (according to the transcriptome data of our lab), respectively, and ABI PRISM 7500 (Applied Biosystems, United States) were used to perform qPCR. Each cDNA sample was run in triplicate, and the assay itself was repeated three times. The 2^-ΔΔCt^ method was used to analyze the data (Chen et al., 2015).

### Subcellular Localization

The *HaGland5* gene without signal peptide was amplified using *HaGland5-*dsp*-*F/*HaGland5-*dsp*-*R primers (Supplementary Table S1), and cloned into pYBA1132 vector (containing GFP). Then the HaGland5-GFP fusion gene, and the vector control expressing GFP alone, were introduced into tobacco leaves through agroinfiltration of EHA105. After 48 h, infiltrated leaves were visualized under a laser confocal fluorescence microscope (Zeiss LSM 880) at an excitation wavelength of 488 nm (Chen et al., 2015).

### Silencing of *HaGland5* by BSMV-HIGS and the *H. avenae* Infection

To analyze the function of HaGland5 of *H. avenae*, we used host-induced gene silencing (HIGS) to silence *HaGland5* of nematode in wheat (Nowara et al., 2010; Chen et al., 2015). The fragment selected to be a silent version of *HaGland5* was confirmed by a BLAST search with NCBI and our transcriptome data of *H. avenae* to ensure the specificity of silencing. Primers *HaGland5-*RNAi-F/*HaGland5-*RNAi-R (Supplementary Table S1) were used to amplify this fragment. Barley stripe mosaic virus-medicated host-induced gene silencing (BSMV-HIGS) and nematode infection assay were conducted as early described (Yuan et al., 2011; Chen et al., 2015). For the infection assay, BSMV:00 and BSMV:*eGFP* were used as negative controls. Each wheat plant was inoculated with 300 pre-J2s, and 35 wheat plants were used for BSMV:00, BSMV:*eGFP* and BSMV:*HaGland5* treatments, respectively. At 7 dpi, the expression level of *HaGland5* in nematodes recovered from the whole wheat roots (*n* = 5 for each treatment) was determined (Chen et al., 2015), and the nematodes in the roots (*n* = 15 for each treatment) were stained and counted (Bybd et al., 1983). At 50 dpi, nematode females on wheat roots and in the soil (*n* = 15 for each treatment) were collected and counted (Chen et al., 2015). Data were analyzed by SPSS v13.0 and differences between the treatment groups were compared by independent-samples *t*-tests (Niu et al., 2016). The experiment was repeated three times independently.

### Generation of Transgenic *A. thaliana* Lines and *H. schachtii* Infection

*HaGland5* and its homologous form without a signal peptide of *H. schachtii* were amplified using the primers 1300-*HaGland5*-F/1300-*HaGland5*-R or 1300-*HsGland5*-F/1300-*HsGland5*-R (Supplementary Table S1), and then cloned into the pSuper1300 vector (with CaMV35S promoter), respectively (Haibian et al., 2010). Then, transgenic plants of *A. thaliana* were obtained using the floral dip method (Clough and Bent, 2010).

For the infection assay, 14-day-old homozygous T3 transgenic plants and the wild-type control of *A. thaliana* were inoculated with 300 pre-J2s of *H. schachtii*. Three weeks post nematode inoculation, the J4s number per root system were counted. There were 20 replicate plants for each plant line. Independent-sample *t*-tests were used to analyze the differences in infection between the treatments. And three independent experiments were performed (Hewezi et al., 2015).

### PTI Assay

For the ROS assay, the ROS burst was detected by luminol-HRP-based chemiluminescence assay. The *HaGland5* gene without signal peptide was amplified by primers 3301-*HaGland5*-F/3301-*HaGland5*-R (Supplementary Table S1), and then *HaGland5*-*GFP* fusion gene, and the vector control expressing GFP alone, were introduced into tobacco leaves by agroinfiltration. After 36 h, the infiltrated leaf discs (4 mm diam.) were collected and incubated overnight in 100 μL of H_2_O in a 96-sample microplate and substituted by 100 μL elicitor master mix (100 μM luminol, 20 μg/ml horseradish peroxidase, 100 nM flg22). ROS production was monitored for 40 min in the microplate reader (Sang and Macho, 2017). The assay was performed three times independently, 24 leaf disks of *N. benthamiana* were collected for each treatment and each time.

For the callose assay, *Arabidopsis* seedlings Col-0 and homozygous T3 transgenic plants either expressing *HaGland5* or *HsGland5* were cultivated on the ½-MS medium for 8 days, and treated with 1 μM of flg22 or distilled water (negative control) for 72 h. Then, *Arabidopsis* seedlings were put in a solution (95% ethanol: acetic acid = 3:1) overnight, then rehydrated 1 h in 70% ethanol, 1 h in 50% ethanol, and 1 h in distilled water, and then treated with 10% NaOH for 1.5 h at 37°C, finally stained in the solution (0.01% aniline blue, 150 mM K_2_HPO_4_, pH 9.5) for at least 1 h. At last, 1–2 cm length root tips (*n* = 12–14) were observed under the microscope (Olympus BX61, Japan) with UV light for callose deposition. Photographs were taken of the root area containing the root elongation zone of *Arabidopsis*. ImageJ software^5^ was used to count callose deposits (Tran et al., 2016). Three independent experiments were conducted.

For detecting the expression level of defense-related genes in transgenic *A. thaliana*, 14-day-old seedlings of Col-0 and homozygous T3 transgenic plants either expressing *HaGland5* or the homozygous form of *HsGland5* were submerged in sterile water containing 10 μM of flg22. After 4 h, 10 mg *A. thaliana* seedlings were prepared for extracting RNA using the TRIzol RNA extraction reagent (Invitrogen, United States). The transcript abundances of *PAD4*, *FRK1*, *CYP81F2, NPR1, WRKY70*, and *PAL4* were detected by RT-qPCR. Each sample reaction was run in triplicate, and independent-sample *t*-tests were used to analyze the differences in transcript abundances. The experiment was repeated three times (Lin et al., 2016).

### Interaction Analysis

*Arabidopsis* seedlings Col-0 and homozygous T3 transgenic plants expressing *HaGland5* cultivated on the ½-MS medium for 14 days were used for the mass spectrometry assay. The whole protein was extracted using a Plant Protein Extraction Kit (CW0885B, CWBIO). After co-immunoprecipitation (Co-IP), the protein complex containing HaGland5-FLAG and its interacting protein partners were captured by FLAG antibody. The interacting proteins were identified by mass spectrometry using Q Exactive (Thermo scientific).

For the Co-IP assay, the *HaGland5* and *AtEMB3003* were cloned into the pYBA1132 (containing GFP tag) and pYBA1143 (containing HA tag), respectively. All constructs were sequenced and introduced into EHA105, and *N. benthamiana* was inoculated with the mixture; GFP was used as the negative control. After 48 h inoculation, the whole *N. benthamiana* proteins were extracted, and the experiment was performed as described before (Koster et al., 2014).

A firefly luciferase (LUC) complementation imaging (LCI) assay was performed following early method (Chen et al., 2008; Yang et al., 2011). *HaGland5*-NLuc (inserted into pCAMBIA-NLuc vector) and Cluc-*AtEMB3003* (inserted into pCAMBIA-CLuc vector) constructs were used for the LCI assay. 39090/XopAF (from Professor Wenxian Sun’s lab, China Agricultural University) was used as the positive control. NLuc/Cluc-*AtEMB3003*, NLuc/CLuc and NLuc/CLuc were used as negative controls. All the resultant constructs were transformed into EHA105. 7-week-old *N. benthamiana* leaves were inoculated with the suspensions. Thereafter, plants were grown at 22°C with a 16 h light/8 h dark cycle in a growth chamber for 60 h. The CCD imaging apparatus (NightSHADE LB985, Berthold) was used to measure the LUC activity.

## Results and Discussion

### Sequence Analysis of *HaGland5* From *H. avenae*

**FIGURE 1:**
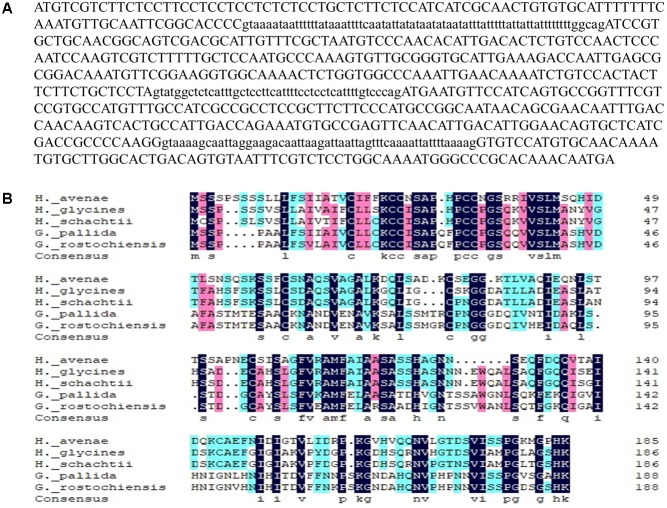
Sequence analysis of *HaGland5* from *Heterodera avenae*. **(A)** Genomic DNA sequence of *HaGland5*. Three introns are shown in lower-case letters. **(B)** Multiple sequence alignment of HaGland5 with homologues from other plant-parasitic nematodes.

Twelve homologues of the *H. avenae* G16B09 family were obtained through a BLAST search of the *H. avenae* transcriptome (Yang et al., 2017) using an *E*-value threshold of 10^-5^ (Thorpe et al., 2014). No domains, motifs, or features could be predicted from the sequences, which were the same as those from *H. glycines* and *G. pallida* (Cotton et al., 2014). The alignment results and phylogenetic tree of these G16B09-like members from *H. avenae* are presented in Supplementary Figure S1. Among these G16B09-like members of *H. avenae*, *HaGland5* was chosen to further characterize the function of this family for the parasitism of *H. avenae*. A 737-bp genomic fragment of *HaGland5* was obtained, consisting of a 561 bp open reading frame, separated by three introns of 73, 47, and 56 bp (Figure 1A). The *HaGland5* cDNA encoded a 186 amino acid protein, with a predicted molecular size of 19.49 kDa that consisted of an N-terminal signal peptide of 26 amino acids (Petersen et al., 2011). According to TMHMM, HaGland5 has no putative transmembrane domain. PSORT analysis showed that HaGland5 has 13.5% chance of being located in the nucleus and 9% chance of being located in the cytoplasm and nucleus.

For the alignment analysis, the homologues with the highest similarity to *HaGland5* from *H. glycines*, *H. schachtii*, *G. pallida*, and *G. rostochiensis* were obtained by a BLAST search against the public genome database or by amplification (Cotton et al., 2014; Eves-van den Akker et al., 2016). An amino acid sequences alignment of these proteins from different nematodes was presented in Figure 1B.

### *HaGland5* Is Expressed in the Dorsal Gland and Is Up-Regulated in Par-J2 Stage of *H. avenae*

**FIGURE 2:**
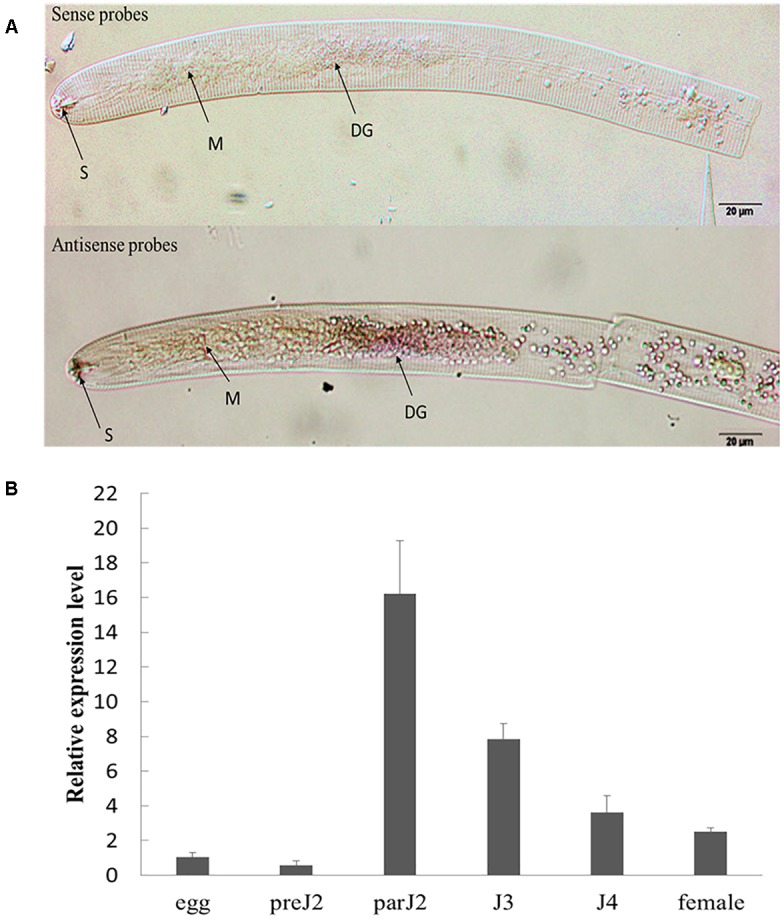
Spatial and developmental expression of *HaGland5* of *Heterodera avenae.* **(A)** A digoxigenin-labeled antisense *HaGland5* cDNA probe localized *HaGland5* transcripts within the dorsal gland cell of the pre-parasitic J2 stages. The dorsal gland (DG), metacorpus (M), and stylet (S) are indicated with arrows. **(B)** Life stage expression of *HaGland5*. The relative expression level of *HaGland5* was quantified using quantitative RT-PCR of six different *H. avenae* life stages: egg, pre-parasitic second-stage juvenile (pre-J2), parasitic second-, third-, fourth-stage juveniles (par-J2, J3, J4), and female. Housekeeping gene *GAPDH-1* was used as a reference gene. The values were calculated using the 2^-ΔΔCt^ method and presented as the fold-change in mRNA level in various nematode developmental stages relative to that of egg. Means ± SD are shown. The results shown are representative of at least three independent experiments.

The spatial expression of *HaGland5* in nematode tissues was determined by *in situ* mRNA hybridization. As reported previously for *H. glycines* and *G. pallida* (Thorpe et al., 2014; Noon et al., 2015), 22 J2s samples showed that hybridization signal was exclusively in the dorsal gland cell (Please also see Supplementary Figure S4 showing the dorsal gland cell’s location of J2) when using the antisense cDNA probe specifically for *HaGland5* (*n* = 25). There was no detected signal when using the sense cDNA probe (Figure 2A).

The developmental expression level of *HaGland5* in *H. avenae* was analyzed by qPCR. The housekeeping gene *GAPDH-1* was used as a reference gene. And the level of expression of *HaGland5* in eggs was set at a value of one, serving as the baseline for examining the relative fold changes in other stages. The results showed that the expression level of *HaGland5* increased in parasitic stages, and reached a peak in the par-J2 stage (Figure 2B). These findings suggest that HaGland5 may promote nematode parasitism in the early stages. The transcriptional level of *HaGland5* was similar with *GrUBCEP12* (Chronis et al., 2013), both expressed more active in the early parasitic stages.

### HaGLAND5 Shows a Nucleocytoplasmic Distribution in Plant Cells

**FIGURE 3:**
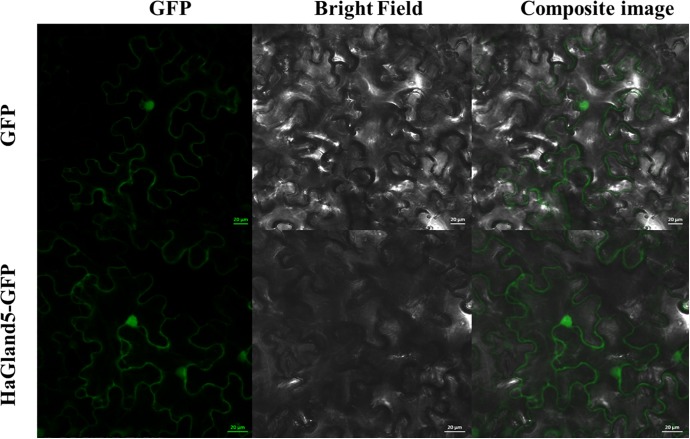
Subcellular location of HaGland5 of *Heterodera avenae*. The subcellular location of HaGland5 in *Nicotiana benthamiana* leaves revealed that 35S:HaGland5-GFP and 35S:GFP were targeted throughout the whole cell. Scale bar = 20 μm.

To examine the subcellular localization of HaGland5 protein in plant cells, a *HaGland5* construct was generated without signal peptide, fused with a GFP protein in the C terminal and 35S promoter (Hewezi et al., 2008). The construct was transiently expressed in *N. benthamiana* leaf cells. The transient expression of both the fusion protein and GFP alone showed nucleocytoplasmic accumulation of the GFP signal (Figure 3), consistent with the PSORT prediction.

### BSMV-HIGS of *HaGland5* Decreases *H. avenae* Pathogenicity

**FIGURE 4:**
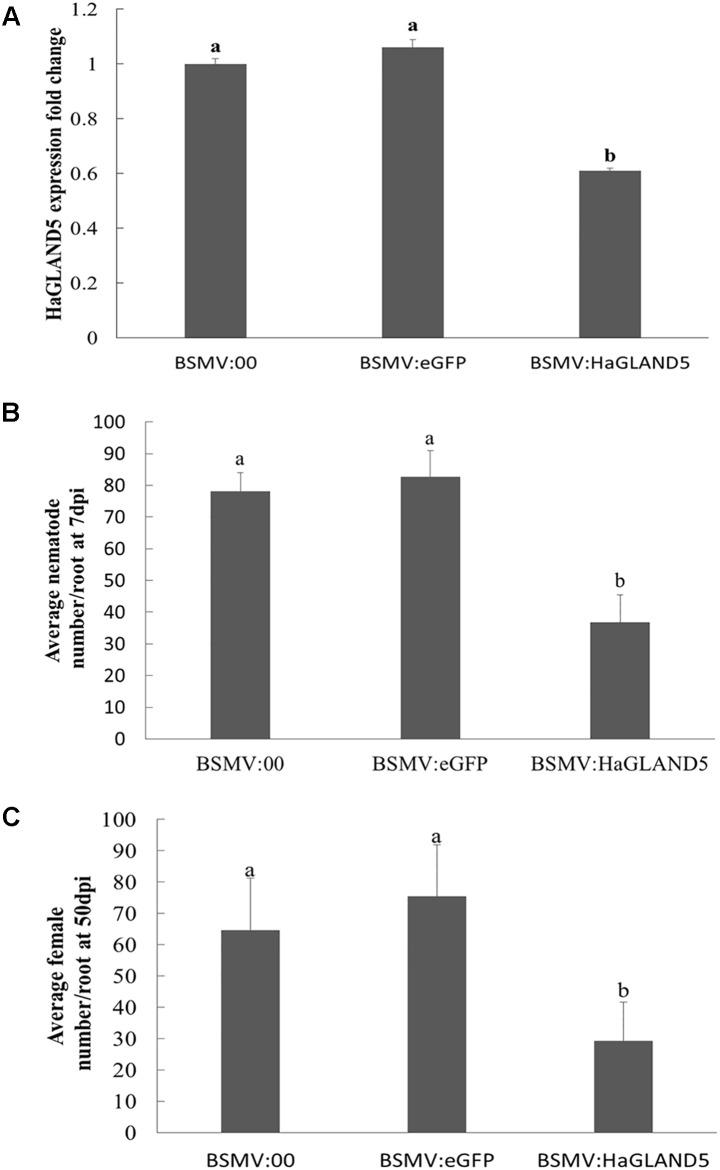
BSMV-HIGS of *HaGland5* decreases *Heterodera avenae* pathogenicity. **(A)** The expression level of *HaGland5* of *H. avenae* collected on wheat inoculated by BSMV:00, BSMV:*eGFP* and BSMV:*HaGland5* at 7 days post-infection. **(B)** The number of nematodes/root in wheat roots at 7 days post-infection. **(C)** The number of females/root at 50 days post-infection in the root surface and soil. Shown values are means ± SD (*n* = 12–15). The independent experiments were repeated three times with consistent results. Columns for the same time point or treatment marked with different letters were significantly different (*P* < 0.05) from each other.

The BSMV-HIGS system emerged from BSMV-VIGS (virus-induced gene silencing). BSMV is a single-stranded RNA virus, and BSMV vectors were efficient as VIGS vehicles in barley and wheat. A construct was obtained to target nucleotides (nt) 121–420 of the *HaGland5* gene (Supplementary Figure S2). BLAST searches showed that this sequence was only present in *H. avenae*, not in wheat and *A. thaliana*. At the same time, a negative construct with *GFP* was also obtained. Our results showed that wheat root length were no significant difference among treatments post 7 days nematode inoculation (Supplementary Figure S3), and the expression of *HaGland5* in nematodes recovered from wheat inoculated by BSMV:*HaGland5* reduced 1.6–1.9-fold, compared with that from the controls BSMV:00 and BSMV:*eGFP* (*P* < 0.05, Figure 4A), indicating that *HaGland5* was effectively silenced. Due to the reducing expression level of *HaGland5*, the number of nematode per plant at 7 dpi from wheat inoculated by BSMV:*HaGland5* was 52.9 or 55.4% lower than that of BSMV:00 and BSMV:*eGFP*, respectively (Figure 4B), and the number of nematode females per plant at 50 dpi from wheat inoculated by BSMV:*HaGland5* was 54.7 or 61.2% lower than that of BSMV:00 and BSMV:*eGFP*, respectively (Figure 4C). These results provide important evidence to suggest the involvement of *HaGland5* in nematode parasitism.

### Over-Expression of *HaGland5* in *A. thaliana* Increases Plant Susceptibility to *H. schachtii*

**FIGURE 5:**
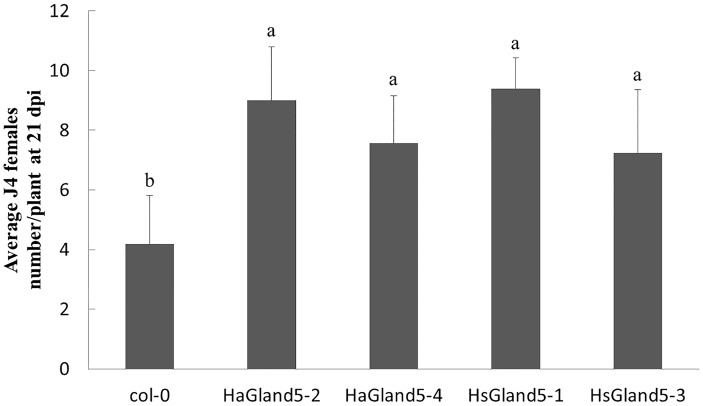
Ectopic-expression of *HaGland5* or *HsGland5* in *A. thaliana* increased plant susceptibility to *Heterodera schachtii*. *A. thaliana* lines expressing *HaGland5* or *HsGland5* tagged with 3 × FLAG, and promoted by 35S, increased the number of nematode females in root compared to wild-type (Col-0) lines. Shown values are means ± SD (*n* = 12–15). The experiments were independently repeated three times with consistent results. Columns for the same time point or treatment marked with different letters were significantly different (*P* < 0.05) from each other.

In the BSMV-HIGS assay, we considered that HaGland5 may promote nematode parasitism. To provide further evidence to support that HaGland5 was closely associated with nematode parasitism, we expressed *HaGland5* in *A. thaliana*, and observed the different phenotypes of the plants to nematode infection. The *H. schachtii*–*A. thaliana* pathosystem was employed, which has been successfully used for analyzing the function of nematode parasitic genes in-depth (Sijmons et al., 1991; Gheysen and Fenoll, 2011). Specifically, HsGland5 from *H. schachtii* shared a 70% sequence identity with HaGland5 in protein level, and it was cloned and used as a homologue control. The root lengths and plant sizes of transgenic *A. thaliana* lines were not significant difference compared with those of wild type by visual inspection. The results showed that either *HaGland5* or *HsGland5* transgenic *A. thaliana* lines were significantly more susceptible to *H. schachtii* than the wild-type control of *A. thaliana* (*P* < 0.05; Figure 5). The results further indicated that HaGland5 plays an important role in nematode parasitism.

Previous research has demonstrated that the *Arabidopsis–H. schachtii* pathosystem is a useful alternative method to explore the effector roles in nematodes, such as *H. glycines*, which do not infect *Arabidopsis*. For instance, HgCBP is a cellulose binding protein of *H. glycines*, to determine the function of CBP, *HsCBP*, an orthologous cDNA from *H. schachtii* was cloned. The roots of transgenic *Arabidopsis* expressing *HsCBP* were longer, and more susceptibility to *H. schachtii*, indicating the crucial function of HgCBP in *H. glycines* (Hewezi et al., 2008). The host range of *H. avenae* is narrow, and the genetic transformation of wheat is time consuming; therefore, the *Arabidopsis–H. schachtii* pathosystem may provide a useful method to explore the function of the effectors of *H. avenae.* Our results showed that *Arabidopsis* plants overexpressing *HaGland5* or *HsGland5* were both more susceptible to *H. schachtii* infection, further supporting that the *Arabidopsis–H. schachtii* pathosystem is a suitable system for exploring the function of effectors of *H. avenae*.

### HaGland5 Suppresses Plant PTI Responses

To further explore the mechanism of HaGland5 in plant defense suppression, we performed PTI suppression assays, including callose deposition, burst of reactive oxygen species, and expression of defense-related genes (Luna et al., 2011; Mendoza, 2011).

**FIGURE 6:**
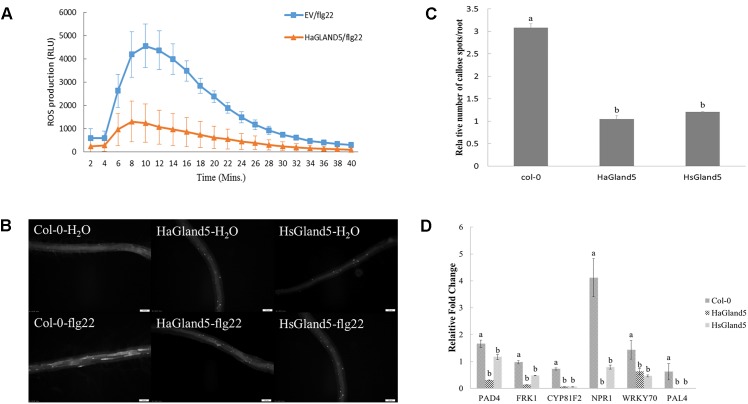
HaGland5 suppresses plant PTI responses. **(A)** *Agrobacterium tumefaciens* strain GV3101 carrying HaGland5-GFP suppressed the ROS production induced by flg22 in *Nicotiana benthamiana* compared with that carrying GFP. **(B,C)** Callose deposition was reduced considerably in the roots of transgenic *Arabidopsis* plants expressing *HaGland5* or *HsGland5* compared with Col-0 plants after being treated with flg22. **(D)** qPCR analysis showed that when challenged with flg22, the expression fold-change of six defense-related marker genes (*PAD4*, *FRK1*, *CYP81F2, NPR1, WRKY70*, and *PAL4*) of the transgenic lines expressing either *HaGland5* or *HsGland5* was significantly lower than that of Col-0 plants. The independent experiments were repeated three times with consistent results.

The ROS burst was an important event for PTI response; therefore, we investigated whether HaGland5 could suppress the ROS production. The results showed that HaGland5 strongly reduced ROS production induced by flg22 compared with the control (Figure 6A). Similarly, callose deposition was considerably reduced in the roots of transgenic *Arabidopsis* plants expressing HaGland5 or HsGland5 compared with Col-0 plants after being treated with flg22 (Figure 6B,C).

qPCR analysis showed that when challenged with flg22, the expression of six defense marker genes (*PAD4*, *FRK1*, *CYP81F2, NPR1, WRKY70*, and *PAL4*) of Col-0 plants boosted strongly higher than that of the distilled water control, while the boost levels of these defense genes of transgenic lines expressing either *HaGland5* or *HsGland5* were much lower than that of the distilled water control (Figure 6D). The *CYP81F2* gene encodes a P450 monooxygenase that is associated with callose deposition (Bednarek et al., 2009); the *FRK1* gene is a fructokinase gene, which is vital in the MAPK signaling pathway (Gonzali et al., 2001). *NPR1, WRKY70, PAD4*, and *PAL4* genes are all associated with the salicylic acid (SA) signal pathway. The *NPR1* gene is an important regulator of the SA-mediated systemic acquired resistance (SAR) pathway, which can activate SA-dependent defense genes (Cao et al., 1997; Wu et al., 2012; Zhu et al., 2017); *WRKY70* can activate SA-induced genes (Li et al., 2013); *PAD4* is a positive regulator that can increase the level of SA (Jirage et al., 1999); *PAL4* is the phenylalanine ammonia-lyase gene, involved in SA biosynthesis (Duan et al., 2015). All these results suggested that HaGland5 can suppress the PTI response in plants.

### HaGland5 Interacts With the AtEMB3003 Protein From *A. thaliana*

To identify potential HaGland5 target proteins in plant cells, we used a Co-IP coupled with mass spectrometry technique (Ning et al., 2014). In Co-IP assay, the protein complex consisting of HaGland5-FLAG and its interacting protein partners were captured by a FLAG antibody. And then the interacting proteins were identified by mass spectrometry. By this method, we obtained six potential HaGland5 target proteins (Supplementary Table S2).

**FIGURE 7:**
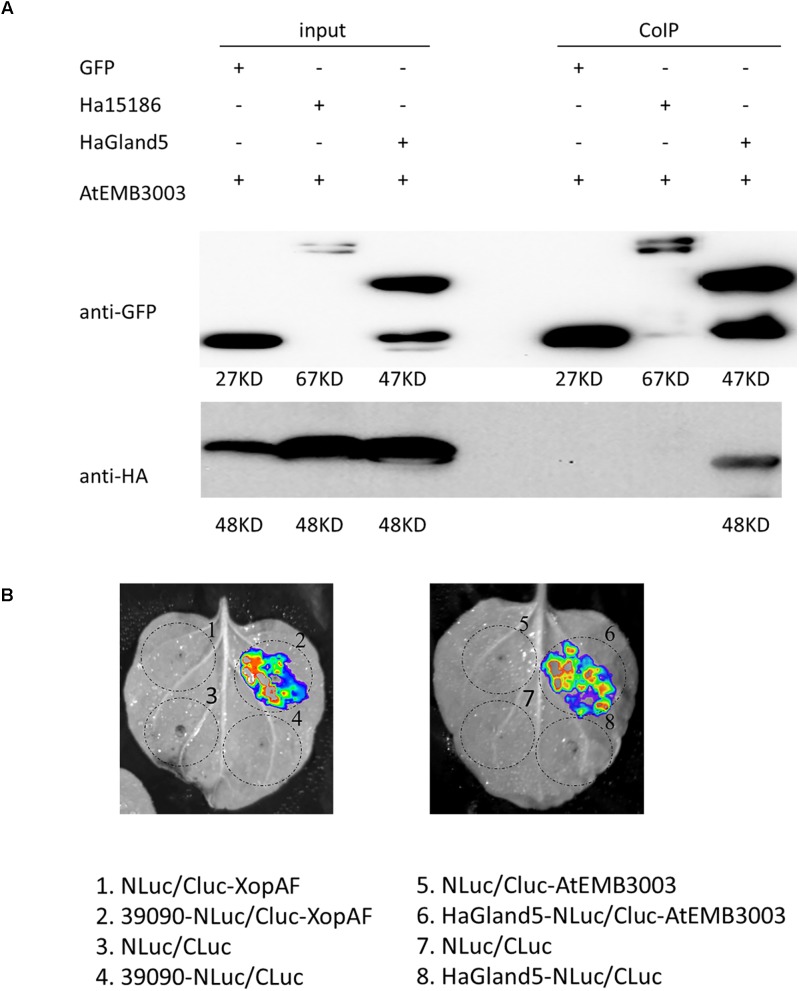
HaGland5 interacts with the AtEMB3003 protein from *A. thaliana*. **(A)** Co-immunoprecipitation (Co-IP) analysis of HaGland5-GFP and AtEMB3003-HA. Western blot analysis confirmed the expression of the input proteins. AtEMB3003-HA was detected only after Co-IP in the sample expressing HaGland5-GFP but not in the sample expressing GFP or Ha15186-GFP. The negative control Ha15186 is another effector of *Heterodera avenae*. **(B)** Luciferase complementation imaging (LCI) assay showed that like positive control 39090/XopAF, HaGland5/AtEMB3003 also had high firefly luciferase (LUC) activity, but no LUC activity in the negative controls (NLuc/Cluc-AtEMB3003, NLuc/CLuc and HaGland5-NLuc/CLuc).

Co-IP assay was employed to examine the interaction between HaGland5 and these candidate proteins. And only AtEMB3003 (At1g34430.1) interacted specifically with HaGland5 (Figure 7A). LCI assay was also used to further confirm their interaction (Chen et al., 2008). The results showed that, like the positive control 39090/XopAF (unpublished), HaGland5/AtEMB3003 also exhibited high LUC activity, but the negative control had no LUC activity (Figure 7B). In conclusion, we confirm that HaGland5 interacts with the AtEMB3003 protein from *A. thaliana.*

AtEMB3003 is an E2 subunit of the plastid PDC (Lin et al., 2003). Unlike mitochondrial counterpart, fatty acid biosynthesis is exclusively in the plastids of plants, and the plastid of PDC can provide the fatty acid precursor, acetyl-CoA (Johnston et al., 1997). Plastid PDC participates in many metabolic pathways, such as glycolysis and tricarboxylic acid cycles. For example, resistance gene-dependent defense signaling in *Arabidopsis* was regulated by Plastidial fatty acid, which could also confer resistance in a SA-independent pathway (Chandra-Shekara et al., 2007). There are many studies showing that fatty acids and their derivatives can participate effectors triggered immune response (Lim et al., 2017). Combined with the results of expression of plant defense-related genes that *NPR1, WRKY70, PAD4*, and *PAL4* genes were all down-regulated in the transgenic lines expressing *HaGland5* or *HsGLAND5*, which are all associated with the SA signal pathway, therefore, we infer that the interaction between HaGland5 and plastid PDC (EMB3003) may be involved in SA-mediated defense pathways.

In summary, we identified a G16B09-like family effector, HaGland5, from the nematode *H. avenae*, which was exclusively expressed in dorsal gland cell of *H. avenae*, and greatly up-regulated in the par-J2 stage. Moreover, HaGland5 could suppress plant defense responses, such as the expression of plant defense-related genes, cell wall callose deposition, and the burst of ROS. Mass spectrometry, Co-IP and LCI assays confirmed that HaGland5 interacted specifically with an *Arabidopsis* pyruvate dehydrogenase subunit (AtEMB3003), which might interfere with the SA signaling pathway and suppress the defense response in plants to promote nematode parasitism.

## Author CONTRIBUTIONS

HJ designed the experiments, and acquired its funding. SY, LP, and YC performed the experiments. DY carried out the bioinformatics analyses. SY, HJ, and QL wrote and revised the manuscript. All authors read and approved the manuscript.

## Conflict of Interest Statement

The authors declare that the research was conducted in the absence of any commercial or financial relationships that could be construed as a potential conflict of interest.

## Footnotes

1 http://www.ncbi.nlm.nih.gov/Structure/cdd/wrpsb.cgi

2 http://www.cbs.dtu.dk/services/SignalP

3 http://www.cbs.dtu.dk/services/TMHMM/

4 https://wolfpsort.hgc.jp/

5 http://imagej.nih.gov/ij/
