# Supplementary material for: Heterodera avenae GLAND5 Effector Interacts With Pyruvate Dehydrogenase Subunit of Plant to Promote Nematode Parasitism
Source: Front Microbiol. 2019 Jun 4;10:1241. doi: 10.3389/fmicb.2019.01241 (PMC6558007; doi:10.3389/fmicb.2019.01241)
Supplement: Supplementary file 1 [file Table_1.DOCX]

**Table S1 Primer sequences used in this study**

| Primer name | Sequence (5'-3') | application |
| --- | --- | --- |
| 3301-HaGland5-F | CCAGCTCCAGCTCCAGGATCCATGGCACCCCATCCGTGCTG | gene amplication  (ROS) |
| 3301-HaGland5-R | TTATGGAGAAAGCTTGGATCCTTGTTTGTGCGGGCCCATTT |  |
| in-situ-HaGland5-F | CTCCATCATCGCAACTGT | in situ hybridization |
| in-situ-HaGland5-R | TGGCACGGACGAAACC |  |
| HaGland5-qRT-F | CTGTCCACTACTTCTTCTGCTCCT | qPCR  (developmental expression) |
| HaGland5-qRT-R | ATTGTTCGCTGTTATTGCCG |  |
| GAPDH-1-qRT-F | AGCGGCACAGAACATCATCC |  |
| GAPDH-1-qRT-R | GGTCCTCCGTGTAGCCCAAA |  |
| PAD4-F | GCCGCTTTCACCGCACTTTGG | qPCR  (the expression level of defense-related gene) |
| PAD4-R | GAGAGATTGGTTTCCGAGCAGAGG |  |
| FRK1-F | TGCAGCGCAAGGACTAGAG |  |
| FRK1-R | ATCTTCGCTTGGAGCTTCTC |  |
| CYP81F2-F | GTGAAAGCACTAGGCGAAGC |  |
| CYP81F2-R | ATCCGTTCCAGCTAGCATCA |  |
| NPR1-F | TGACTAGCCTCGAGCCTGAC |  |
| NPR1-R | GCAAGAGTCTCACCGACGAC |  |
| WRKY70-F | CATACATAGGAAACCACACG |  |
| WRKY70-R | CTCCAAACACCATGAGATCC |  |
| PAL4-F | ATCAGCAGTGAGTCAGGTGG |  |
| PAL4-R | CTTGAGACATTCCAACAACG |  |
| HaGland5-dsp-F | CGCTCTAGAACTAGTGGATCCATGGCACCCCATCCGTGCTG | gene amplication  (subcellular localization) |
| HaGland5-dsp-R | GGGCCCCCCCTCGAGGTCGACTTGTTTGTGCGGGCCCATTT |  |
| HaGland5-RNAi-F | AAGGAAGTTTAAGTTTCGCTAATGTCCCAAC | RNAi |
| HaGland5-RNAi-R | AACCACCACCACCGTAATGGCAGTGACTTGTTGG |  |
| Q-HIG-HaGLAND5-F | TGACCAGAAATGTGCCGAGTT | qPCR (the expression level of HaGland5 in BSMV-HIGS assay) |
| Q-HIG-HaGLAND5-R | TTGCCAGGAGACGAAATTACA |  |
| 1300- HaGLAND5-F | CAAATCGACTCTAGAAAGCTTATATGGCACCCCATCCGTGCTG | gene amplication  ( transgenic *A. thaliana* ) |
| 1300- HaGLAND5-R | GTCTTTGTAGTCCATGGTACCTTGTTTGTGCGGGCCCATTT |  |
| 1300- HaGLAND5-F | CAAATCGACTCTAGAAAGCTTATATGGCACCGCATCCGT |  |
| 1300- HaGLAND5-R | GTCTTTGTAGTCCATGGTACCTTGTTTGTGTGAGCCGGTC |  |
| HgGland5-F | ATGTCTTCTCCTTCTTCGTCC | gene amplication  (HsGland5 gene) |
| HgGland5-R | TCATTGTTTGTGTGAGCCG |  |

**Table S2 The potential interact proteins obtained by MS technique and with the information accession numbers and description of the proteins.**

|  | **Accession** | **Description** |
| --- | --- | --- |
| **1** | **AT1G34430.1** | **EMB3003 \| Dihydrolipoyllysine-residue acetyltransferase component 5 of pyruvate dehydrogenase complex, chloroplastic** |
| 2 | AT1G01090.1 | PDH-E1 ALPHA \| pyruvate dehydrogenase E1 alpha |
| 3 | AT4G02520.1 | ATGSTF2, ATPM24.1, ATPM24, GST2, GSTF2 \| glutathione S-transferase PHI 2 |
| 4 | AT3G55550.1 | Concanavalin A-like lectin protein kinase family protein |
| 5 | AT3G21180.1 | ACA9, ATACA9 \| autoinhibited Ca(2+)-ATPase 9 |
| 6 | AT3G13930.1 | Dihydrolipoamide acetyltransferase, long form protein |


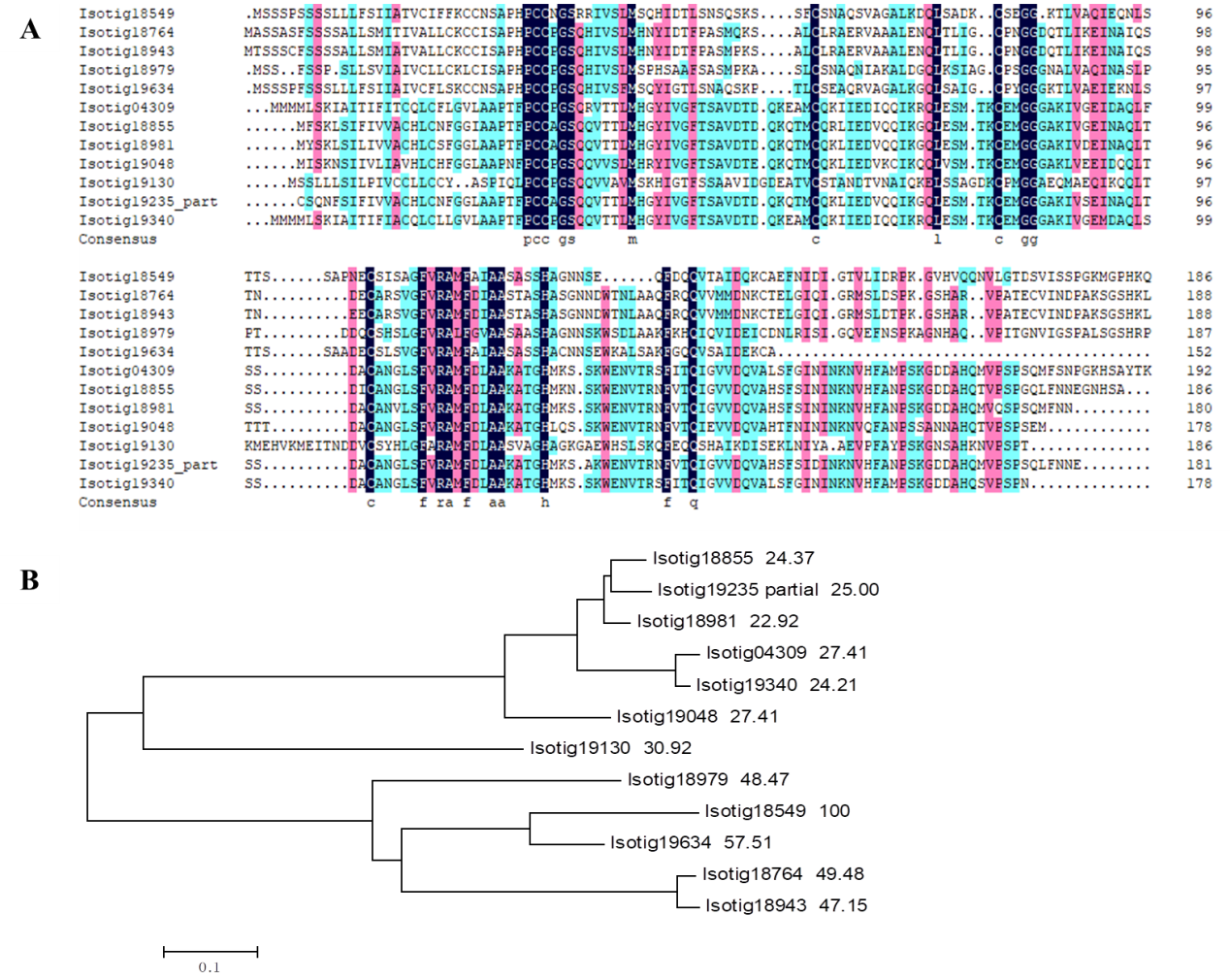


**Fig S1.** Alignment and phylogenetic tree of 12 homologues of the G16B09 family from *Heterodera avenae.* (A) Alignment of 12 homologues of the G16B09 family from *Heterodera avenae* by DNAMAN*.* (B) Phylogenetic tree of 12 homologues of the G16B09 family from *Heterodera avenae* by MEGA6.0 *.* The members were listed using transcriptome identification numbers, the similarity with *HaGland5* (isotig18549) is indicated after every member.


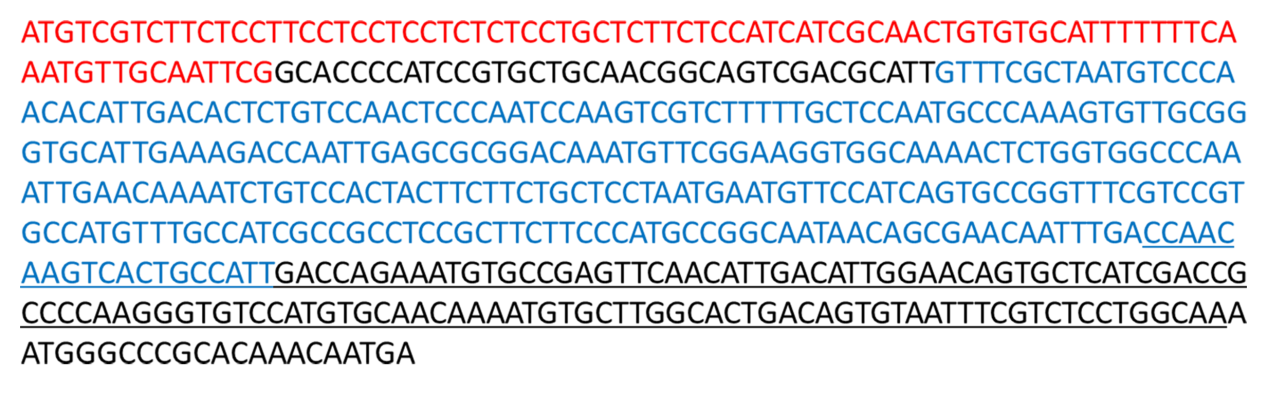


**Fig S2.** The nucleotide sequence of the *HaGland5* gene. The red characters (1–84 bp): signal peptide; the blue characters (121–420 bp): silent fragment of *HaGland5* of the BSMV-HIGS assay; the characters underlined (401–539 bp): the fragment that was used to detect the expression of the *HaGland5* gene by qRT-PCR in the BSMV-HIGS assay.


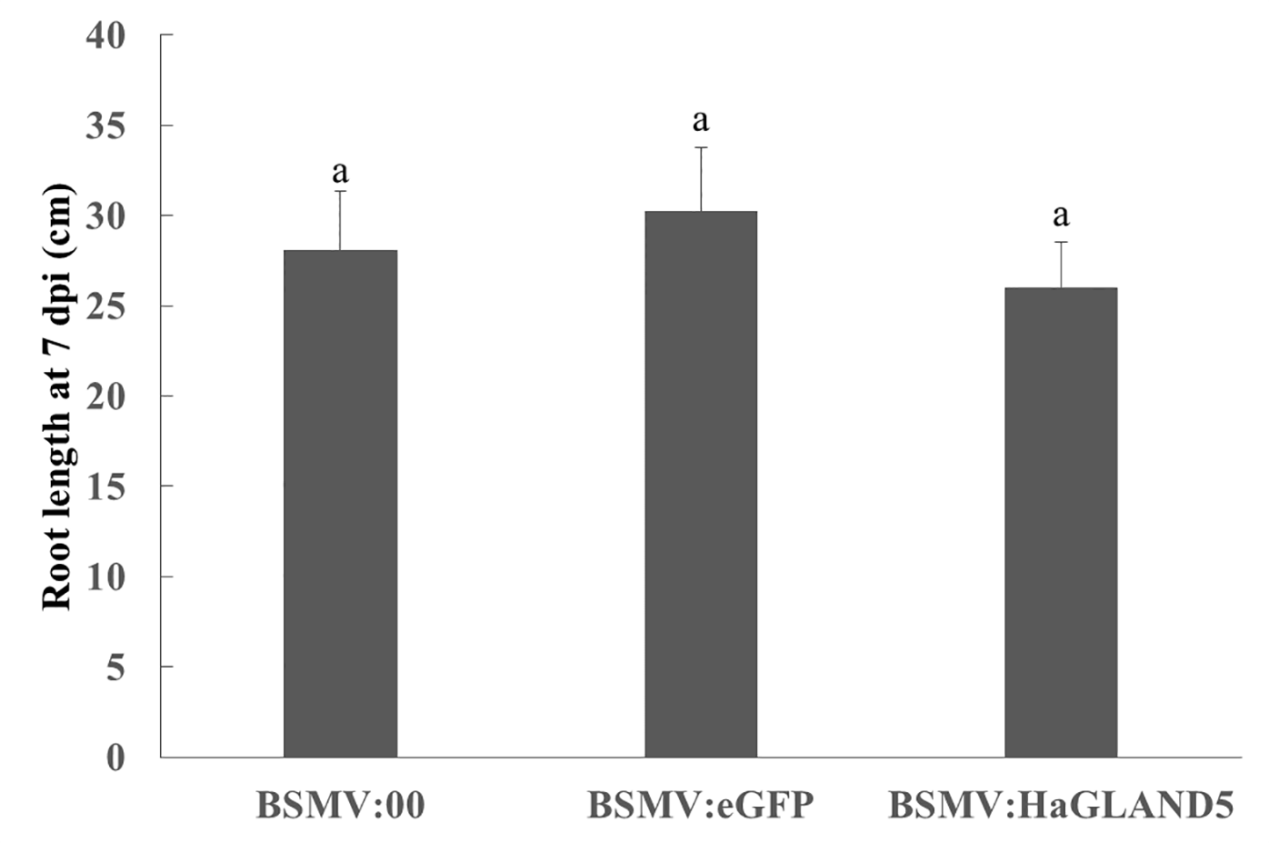


**Figure S3.** The root length of wheat at 7 days post-infection.


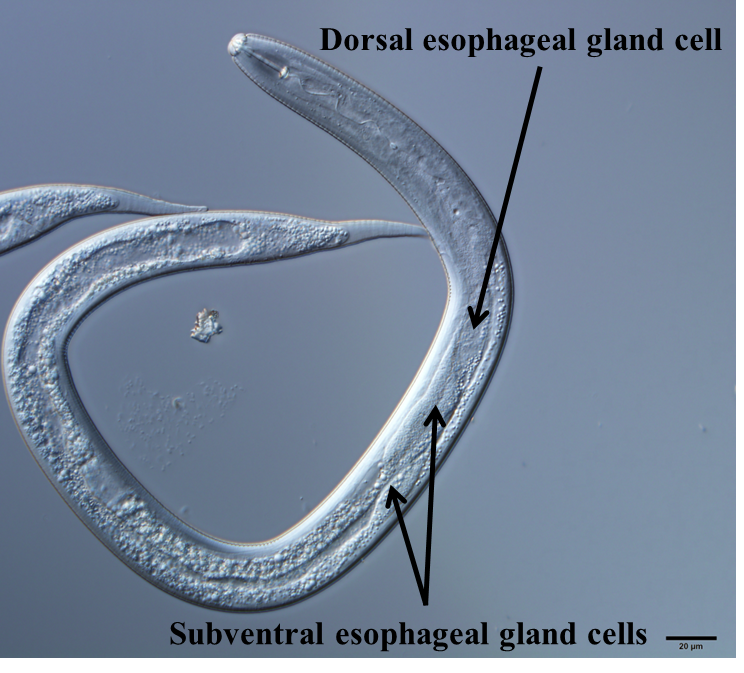


Fig S4. The light micrograph of J2 *H. avenae* showed the position of one dorsal esophageal gland cell and two subventral esophageal gland cells.
